# Melatonin and melatonin agonists to prevent and treat delirium in critical illness: a systematic review protocol

**DOI:** 10.1186/s13643-016-0378-2

**Published:** 2016-11-24

**Authors:** Jennifer Foster, Lisa D. Burry, Lehana Thabane, Karen Choong, Kusum Menon, Mark Duffett, Alexandra Cheung, Melanie Guenette, Timothy Chimunda, Louise Rose

**Affiliations:** 1 Department of Paediatrics, Schulich School of Medicine & Dentistry, Western University, London, Canada; 2 IWK Health Centre, Dalhousie University, 5850/5980 University Avenue, Halifax, NS B3K 6R8 Canada; 3 Division of Children’s Health & Therapeutics, Children’s Health Research Institute, London, Canada; 4 Leslie Dan Faculty of Pharmacy, University of Toronto, Toronto, ON Canada; 5 Department of Pharmacy, Mount Sinai Hospital, 600 University Avenue, Room 18-377, Toronto, ON M5G1X5 Canada; 6 Department of Clinical Epidemiology and Biostatistics, McMaster University, Hamilton, Canada; 7 Biostatistics Unit, Centre for Evaluative Medicine, St. Joseph’s Healthcare, Hamilton, ON Canada; 8 Population Health Research Institute, Hamilton Health Sciences, Hamilton, ON Canada; 9 Department of Anesthesia, McMaster University, Hamilton, ON Canada; 10 Department of Pediatrics, McMaster University, Hamilton, ON Canada; 11 Pediatric Critical Care Unit, McMaster Children’s Hospital, 1200 Main Street West, Hamilton, ON L8N 3Z5 Canada; 12 Children’s Hospital of Eastern Ontario Research Institute, 401 Smyth Road, Ottawa, ON K1H 8L1 Canada; 13 Division of Critical Care, Department of Pediatrics, Children’s Hospital of Eastern Ontario, Ottawa, ON Canada; 14 Departments of Pediatrics and Epidemiology and Community Medicine, University of Ottawa, Ottawa, ON Canada; 15 Department of Anesthesia, University of Toronto, Toronto, ON Canada; 16 School of Medicine, University of Queensland, Brisbane, QLD 4072 Australia; 17 Department of Critical Care Medicine, Sunnybrook Health Sciences Centre, 2075 Bayview Avenue, Toronto, ON M4N 3M5 Canada; 18 Evaluative Clinical Sciences, Trauma, Emergency & Critical Care Research Program, Sunnybrook Research Institute, Toronto, ON Canada; 19 Lawrence S. Bloomberg Faculty of Nursing, University of Toronto, Toronto, ON Canada; 20 Institute for Clinical Evaluative Sciences, Toronto, ON Canada; 21 Provincial Centre of Weaning Excellence, Toronto East General Hospital, Toronto, ON Canada

**Keywords:** Melatonin, Melatonin agonists, Delirium, Prevention, Treatment, Intensive care, Systematic review

## Abstract

**Background:**

Delirium is a syndrome characterized by acute fluctuations and alterations in attention and arousal. Critically ill patients are at particularly high risk, and those that develop delirium are more likely to experience poor clinical outcomes such as prolonged duration of ICU and hospital length of stay, and increased mortality. Melatonin and melatonin agonists (MMA) have the potential to decrease the incidence and severity of delirium through their hypnotic and sedative-sparing effects, thus improving health-related outcomes. The objective of this review is to synthesize the available evidence pertaining to the efficacy and safety of MMA for the prevention and treatment of ICU delirium.

**Methods:**

We will search Ovid MEDLINE, Web of Science, EMBASE, PsycINFO, the Cochrane Central Register of Controlled Trials (CENTRAL), and CINAHL to identify studies evaluating MMA in critically ill populations. We will also search http://apps.who.int/trialsearch for ongoing and unpublished studies and PROSPERO for registered reviews. We will not impose restrictions on language, date, or journal of publication. Authors will independently screen for eligible studies using pre-defined criteria; data extraction from eligible studies will be performed in duplicate. The Cochrane Risk of Bias Scale and the Newcastle-Ottawa Scale will be used to assess the risk of bias and quality of randomized and non-randomized studies, respectively. Our primary outcome of interest is delirium incidence, and secondary outcomes include duration of delirium, number of delirium- and coma-free days, use of physical and chemical (e.g., antipsychotics or benzodiazepines) restraints, duration of mechanical ventilation, ICU and hospital length of stay, mortality, long-term neurocognitive outcomes, hospital discharge disposition, and adverse events.

We will use Review Manager (RevMan) to pool effect estimates from included studies. We will present results as relative risks with 95% confidence intervals for dichotomous outcomes and as mean differences, or standardized mean differences, for continuous outcomes.

**Discussion:**

Current guidelines make no pharmacological recommendations for either the prevention or treatment of ICU delirium. This systematic review will synthesize the available evidence on the efficacy and safety of MMA for this purpose, thus potentially informing clinical decision-making and improving patient outcomes.

**Systematic review registration:**

PROSPERO CRD42015024713

**Electronic supplementary material:**

The online version of this article (doi:10.1186/s13643-016-0378-2) contains supplementary material, which is available to authorized users.

## Background

Delirium is a syndrome characterized by acute fluctuations and alterations in attention and arousal [1]. Critically ill patients in particular are at increased risk, with reported rates of up to 80% in those who are mechanically ventilated [2–4]. Delirium is associated with increased mortality, prolonged duration of intensive care unit (ICU) and hospital length of stay, greater risk of unplanned extubation and device removal, and long-term cognitive sequelae [5].

The pathophysiology of ICU delirium remains unclear. It is hypothesized that gamma-aminobutyric acid or dopamine neurotransmitter pathways are involved in its development and that cytokine passage across the blood-brain-barrier aggravates the condition [5]. Although disturbances of the sleep-wake cycle are not diagnostic of delirium, changes in sleep patterns are incorporated into delirium screening tools, and studies indicate that sleep changes occur in >75% of delirious patients [6]. Critically ill patients, irrespective of delirium status, display abnormal sleep architecture [7, 8]. Sedative drugs such as benzodiazepines, propofol, α_2_-agonists, and opioids, which are frequently administered in the context of critical care, decrease rapid eye movement, the restorative component of sleep [9, 10].

Melatonin is a neuro-hormone produced by the pineal gland during hours of darkness. It has multiple biological effects, most notably on the regulation and synchronization of circadian rhythms [11]. Melatonin works as a hypnotic by accelerating sleep initiation and improving sleep maintenance and efficiency. It exerts other diverse regulatory functions beyond those on sleep; for example, melatonin has anti-excitatory effects on the central nervous system (e.g., vasomotor control), plays an important role in immunomodulation, and has anti-inflammatory [12–17] and anti-oxidative properties [12, 15, 17].

Pineal function and diurnal variation of melatonin vary greatly between individuals; however, these functions are affected by age, cognitive impairment, and several factors common in critical illness [8, 11, 18–26]. Disturbances in circadian melatonin secretion have been described in a number of patient populations, including general medicine, intensive care, and post-operative diagnosed with delirium [24, 27–32]. Specifically, early studies of melatonin secretion in both critically ill children and adults have demonstrated disturbed diurnal rhythms, with loss of normal day-night variation in melatonin secretion [8, 24, 28, 29, 33–36]. Total daily melatonin excretion and serum concentrations have also been shown to be abnormal, although not all studies show similar results [37]. Coupled with the complex pathophysiology of underlying critical illness [4, 38], and the gamut of iatrogenic interventions that can precipitate delirium, critically ill patients are understandably at increased risk [39].

While the effects of abnormal melatonin secretion in the context of critical illness are not fully understood, it is plausible that a relationship exists with the development of delirium. Some studies have shown low melatonin concentrations to be associated with ICU delirium [28, 32], and administration of enteral melatonin has been shown to reduce the incidence of delirium in perioperative and geriatric patient populations [40–42].

At this time, exogenous melatonin is most commonly used for jet lag [43] and primary sleep disorders [44]. Melatonin receptor agonists (e.g., ramelteon) have been developed with the intent of providing potent agonism at the melatonin receptor, while conferring a longer half-life and better absorption than orally administered melatonin. In contrast to conventionally used sleep-aids and sedatives (e.g., benzodiazepines), melatonin and melatonin agonists (MMA) have no potential for abuse, display fewer carry-over effects, and result in minimal cognitive impairment [45]. As a result, and in addition to their direct effect on sleep architecture [46], MMA may be sedative-sparing [47], therefore providing additional use in the prevention and treatment of delirium [28, 32, 40, 48].

While MMA are currently marketed primarily to treat sleep disorders, their use is increasing in critically ill patients, despite unclear evidence supporting their safety and efficacy in this population [45, 49]. A systematic exploration on the topic is therefore warranted, and we propose herein a comprehensive synthesis of existing data of all studies employing MMA in critically ill patients for the prevention and treatment of delirium.

## Methods and design

This protocol was prepared using the Preferred Reporting Items for Systematic Reviews and Meta-Analyses Protocol (PRISMA-P) [50, 51] guidelines. A PRISMA-P checklist (Additional file 1) was completed, and the protocol for this systematic review was registered on the PROSPERO International Prospective Register of Systematic Reviews (CRD42015024713).

### Data sources and search strategy

A MEDLINE electronic search strategy (Additional file 2) was developed through an iterative process with the assistance of an experienced information specialist. The strategy was adapted for other databases and reviewed by another information specialist using the Peer Review for Electronic Search Strategies (PRESS) [52, 53]. The following electronic databases were searched from 1960, roughly corresponding to the time of melatonin discovery (i.e., 1958) and inception of ICUs, to June 2016: Ovid MEDLINE, Web of Science, EMBASE, PsycINFO, Cochrane Central Register of Controlled Trials (CENTRAL), CINAHL, and PROSPERO (http://www.crd.york.ac.uk/PROSPERO/). No restrictions on language, date, or journal of publication will be imposed. A combination of MeSH terms (e.g., intensive care unit, melatonin, melatonin receptor agonist, delirium) and keywords (e.g., intensive care unit, ramelteon) will be applied. Unpublished and ongoing trials will be identified using the term “melatonin” on the International Clinical Trials Registry Platform (http://apps.who.int/trialsearch). Lastly, the reference lists of all relevant studies and review articles will be searched for studies not identified by electronic searches.

### Study eligibility criteria

Randomized controlled trials (RCTs) (using individual or cluster randomization), quasi-randomized controlled trials, and non-randomized studies evaluating MMA for the prevention or treatment of delirium in critically ill patients will be included. Non-randomized controlled studies will include those of the following design, so long as they contain an identified control group: pre- and post, historical control, or cohort (retrospective or prospective). Case studies and case series will be excluded. No a priori restrictions on methodological quality will be imposed.

#### Population

Our population of interest is critically ill patients admitted to an ICU of any type (e.g., cardiac, burn, medical, surgical, trauma, neurologic, mixed). Studies of adults (aged 18 years or older) and children (aged 6 months to 18 years) will be included, as will those treating patients in units providing intermediate care between that of an ICU and an inpatient ward (i.e., step-down/up unit). Such studies will be included because certain countries, smaller centers, or pediatric units in particular do not have standalone intermediate care units that are separate from the ICU.

#### Interventions

Studies evaluating MMA in the prevention or treatment of ICU delirium will be included. Interventions may comprise, but are not limited to, melatonin, ramelteon, agomelatine, tasimelteon, or TIK 301. No restrictions in terms of dose, frequency, route, or duration of administration will be imposed. Studies in which MMA were administered (a) once delirium develops (i.e., treatment), in the ICU; (b) prior to delirium development (i.e., prevention), in the ICU; and (c) prior to delirium development (i.e., prevention), before admission to ICU (e.g., as pre-medication before surgery, in emergency patients with expected transfer to ICU, at time of delivery in premature infants), will be included.

#### Comparators

Studies for the prevention or treatment of delirium in critically ill patients will be included if they compare MMA to placebo or any alternative pharmacological (e.g., benzodiazepine, opioid, antipsychotic, hypnotic, antihistamine, antidepressant, HMG-CoA reductase inhibitor) or non-pharmacological intervention (e.g., noise reduction, early mobilization, music therapy, acupuncture, multi-component therapies).

#### Outcomes

The primary outcome of interest is delirium incidence, defined as at least one episode of delirium experienced during ICU admission and identified using either a validated delirium screening tool (e.g., Confusion Assessment Method for the ICU (CAM-ICU) [54], pediatric Confusion Assessment Method for the ICU (p-CAM-ICU) [55], Intensive Care Delirium Screening Checklist (ICDSC) [56], Neelon and Champagne (NEECHAM) [57] Confusion Scale, Cornell Assessment of Pediatric Delirium (CAP-D) [58]) or assessment by a trained individual (e.g., psychiatrist) using Diagnostic and Statistical Manual of Mental Disorders (DSM) [1, 59] criteria.

Secondary outcomes are total duration of delirium, number of delirium- and coma-free days, use of physical and chemical (e.g., antipsychotics or benzodiazepines) restraints, duration of mechanical ventilation, ICU and hospital length of stay, mortality, long-term neurocognitive outcomes, hospital discharge disposition, and incidence and description of all adverse events (e.g., headache, excessive somnolence, vertigo, abdominal cramping, depression, nausea, vivid dreams and/or nightmares, rash, device removal, need for re-intubation).

### Screening and data extraction

Two authors (JF, LB) will independently screen search results against eligibility criteria to identify relevant trials. Prior to this, a study screening form will be developed and a calibration exercise will be performed to ensure inter-rater reliability in the application of eligibility criteria. Reference management software package EndNote X7 will be used. Two authors (JF, LB) will assess the full text of any title or abstract selected using the screening form to confirm inclusion. Any disagreement will be referred to a third author (LR), who will serve as an independent arbiter. Where necessary, study authors will be contacted to clarify or obtain additional information in order to confirm study inclusion. The Cochrane checklist for assessment of non-randomized studies [60] will be used to categorize study design for study inclusion. The search strategy and study selection process will be documented using a PRISMA flow diagram [61].

Two authors (AC, TC) will independently extract data from included studies. Data extraction will be done in duplicate, using a standardized extraction form that will be developed and piloted on a sample of three studies to ensure capture of all relevant data. Data related to study design and setting, patient demographics (e.g., age, gender, severity of illness score, reasons for ICU admission), verbatim descriptions of delirium prevention and/or treatment, and of comparator arm interventions, any co-interventions that may influence the development of, or be administered in the context of delirium (e.g., avoidance of medications that may be associated with delirium, such as benzodiazepines), as well as data on outcomes of interest, will be collected.

In the event of missing or unclear data, a maximum of three attempts will be made to contact study authors for clarification. Any disagreements pertaining to extracted data will be discussed among data extractors, and, where necessary, discrepancies will be resolved by a third author (JF).

### Study risk of bias assessment

Two authors (AC, TC) will independently assess the risk of bias for each study, and a third author (JF) will confirm the final assessment where any disagreement occurs. If insufficient detail is provided to confidently assess risk of bias, a maximum of three attempts will be made to contact study authors for clarification.

The assessments of RCTs will be performed according to domain-based evaluations published by The Cochrane Collaboration [60]. These domains include the following: (1) random sequence generation (i.e., selection bias), (2) allocation concealment (i.e., selection bias), (3) blinding of participants and personnel (i.e., performance bias), (4) blinding of outcomes assessment (i.e., detection bias), (5) incomplete outcome data (i.e., attrition bias), (6) selective reporting, and (7) other bias (e.g., source of funding).

For each domain, the risk of bias will be assessed as “low,” “high,” or “unclear.” An unclear risk will be assigned for a domain if insufficient detail is reported and cannot be obtained from study authors, or if what happened in the study is known, but its contribution to the risk of bias is unknown or unclear. Each endpoint and the risk of bias will be assessed individually to generate an overall score. After the assignment of risk of bias, studies will be classified according to the following categories:

1. Low risk: studies where all domains are considered to be at “low” risk of bias
2. High risk: studies where one or more domains are considered to be at “high” risk of bias
3. Unclear risk: studies where one or more domain(s) have “unclear” risk of bias

Quality assessment of non-randomized studies will be performed using the Newcastle-Ottawa scale [62]. Using this tool, each study is judged on eight items falling into three categories: (1) selection of study groups, (2) comparability of groups, and (3) ascertainment of exposure or outcome of interest. The scale permits a semi-quantitative assessment of study quality, which can then be incorporated into the interpretation of meta-analytic results.

### Approach to evidence synthesis

Review Manager (RevMan 5.3) [63] software will be used to enter all study data. Study characteristics will be summarized using frequencies and percentages for categorical variables and means and standard deviations, or median and interquartile ranges, for continuous variables, depending on what is reported.

Data on primary and secondary outcomes will be analyzed in aggregate. Binary outcomes will be expressed in terms of risk ratios with 95% confidence intervals. For continuous variables, a pooled difference of means with 95% confidence intervals will be calculated using a DerSimonian Laird random-effects model [64]. Where means (with standard deviations) are not available, these will be approximated using medians (with interquartile ranges) according to the method described by Hozo and colleagues [65], and approximate standard deviations will be calculated from interquartile ranges [60]. If required, skewed data will be log-transformed. A two-sided *P* value <0.05 will be considered to be significant. Review Manager (RevMan 5.3) [63] software will be used to produce forest plots and conduct meta-analyses. In the event that a meta-analysis is not possible (e.g., reporting of outcomes does not permit combing the data), a qualitative synthesis will be provided.

Where possible, the unit of analysis will be the individual participants in each trial arm. In the case of multi-arm studies (e.g., multiple doses of melatonin compared to placebo), the combination of groups to create a single pair-wise comparison will be attempted, as recommended by the Cochrane Handbook for Systematic Reviews of Interventions [60].

In the case of missing data, study authors will be contacted a maximum of three times to inquire about obtaining data. If data remain unavailable, a qualitative description of these studies will be provided, and the potential impact of such missing data will be addressed in the discussion section of the manuscript.

The degree of statistical heterogeneity will be evaluated from forest plots, using chi square tests (*P* < 0.05 represents significant heterogeneity) and the *I* ^2^ statistic (*I* ^2^ > 50% indicates moderate to substantial heterogeneity). Where the *I* ^2^ statistic is >50%, clinical and methodological sources of heterogeneity will be qualitatively assessed, such as through patient demographics (including age and underlying disease), drug choice (melatonin versus melatonin receptor agonist), timing of drug initiation during critical illness, dosing strategies and delivery route (oral or intravenous), and management of room lighting.

Publication bias will be assessed through the construction of a funnel plot of all included studies. For continuous outcomes, the Egger regression [66] and Macaskill [67, 68] tests will be used to detect funnel plot asymmetry where more than ten trials are available. For dichotomous outcomes, the arcsine test will be used [69].

### Subgroup and sensitivity analyses

The following additional subgroup analyses will be performed, where sufficient data exist:

- Age: children, defined as age of 6 months to 18 years, and adults, defined as ≥18 years
- Drug type: melatonin and melatonin agonists, such as tasimelteon and ramelteon
- Comparison group: standard unit-specific care, non-pharmacological interventions, or antipsychotic agent
- Study design: individual versus cluster randomization

We will conduct a sensitivity analysis for the primary outcome of delirium incidence, excluding studies with high risk of bias. Post hoc sensitivity analysis will be performed, where appropriate.

### Reporting of review findings

We will use the Grading of Recommendations Assessment, Development and Evaluation (GRADE) approach [70] to assess the quality of evidence for the primary outcome as well as the following secondary outcomes: duration of delirium, duration of mechanical ventilation, length of ICU and hospital stay, and mortality. A final overall quality of evidence will be summarized in a summary of findings table.

### Dissemination of findings

We will communicate the findings of this systematic review via publication in a peer-reviewed journal. We will present our results at local and national forums in order to relay our findings to clinicians and researchers of the critical care community.

## Discussion

Delirium is a significant health concern in critically ill patients of all ages. At this time, however, no evidence exists to support the use of any particular pharmacological intervention in either its prevention or treatment. Adult critical care guidelines [71] remain conservative in their suggested approaches to patient management, emphasizing the overall lack of good quality evidence and promoting the use of non-pharmacological approaches. Within the pediatric population, there is such a paucity of research in both pharmacological and non-pharmacological approaches that no formal guidelines on delirium management exist at this time. Given the established negative sequelae of delirium and the added vulnerability of the critical care population, it is imperative that safe and effective prevention and treatment strategies be identified. Our proposed systematic review will address the existing knowledge gap regarding the efficacy and safety, in particular, of MMA for the prevention and treatment of delirium in critically ill patients. A thorough synthesis of the available evidence will permit identification of evidence gaps, therefore informing clinical decision-making and guiding future research activities.

## Additional files

Additional file 1: Preferred Reporting Items for Systematic Reviews and Meta-Analyses Protocol (PRISMA-P). PRISMA-P items and their respective line locations within the manuscript. (DOCX 135 kb)
Additional file 2: Search strategy. Search strategy, including all MeSH terms and key words used. (DOCX 65 kb)

## Abbreviations

ICU: Intensive care unit
MMA: Melatonin and melatonin agonists
PRISMA: Preferred Reporting Items for Systematic Reviews and Meta-Analyses
RCT: Randomized controlled trial
