# Supplementary material for: Melatonin and melatonin agonists to prevent and treat delirium in critical illness: a systematic review protocol
Source: Syst Rev. 2016 Nov 24;5:199. doi: 10.1186/s13643-016-0378-2 (PMC5122010; doi:10.1186/s13643-016-0378-2)
Supplement: Additional file 2: — Search strategy. Search strategy, including all MeSH terms and key words used. (DOCX 65 kb) [file 13643_2016_378_MOESM2_ESM.docx]

**Additional file 2: Search strategy run June 2016**

**Concept 1 Melatonin & synonyms (MeSH OR textwords)**

melatonin*

tasimelteon

ramelteon

S-20098

S20098

TIK-301

TIK301

circadin

LY-15635

rozerem

hetlioz

valdoxan

thymanax

melitor

*Melovine*

*“5-methoxy-n-acetyltryptamine”*

*“n-acetyl-5-methoxytryptamine”*

*“vec-162”*

*vec162*

*“tak-375”*

*tak375*

OR MeSH:

Melatonin[Mesh]

Receptors, Melatonin[Mesh]

tasimelteon [Supplementary Concept]

ramelteon [Supplementary Concept]

S 20098 [Supplementary Concept]

**Concept 2 Delirium & synonyms (MeSH OR textwords)**

cap-d

"cornell-assessment-of-pediatric-delirium"

neecham*

nee-cham*

"neelon-and-champagne-confusion-scale"

ICDSC

"intensive-care-delirium-screening-checklist”

“intensive-care-delirium-screening-checklists”

"pediatric-confusion-assessment-for-the-icu"

“p-cam-icu”

“cam-icu”

"confusion-assessment-method-for-the-icu"

delirium*

delirious

disorientat*

confus*

agitat*

inattentive*

hallucinat*

*“acute-brain-dysfunction”*

*“acute-brain-failure”*

*“acute-brain-syndrome”*

*“clouded-consciousness”*

*“clouded-state”*

*“clouded-states”*

*“intensive-care-psychoses”*

*“intensive-care-psychosis”*

*“cognitive-dysfunction”*

*“psycho-organic-syndrome”*

*“psycho-organic-syndromes”*

*“organic-psychosyndrome”*

*“organic-psycho-syndrome”*

OR MeSH:

Delirium[Mesh]

Psychomotor Agitation[Mesh]

Hallucinations[Mesh]

Confusion"[Mesh]

*Psychosis, substance-induced*

**Concept 3 ICU or synonyms:**

operat*[ti]

intraoperat*[ti]

surger*[ti]

postop*

periop*

"operating-room"

“operating-rooms”

"intensive-care"

"intensive-illness"

“intensive-illnesses”

“intensively-ill”

“intensive-care-illness”

“intensive-care-illnesses”

"critical-care"

“critical-ill”

“critical-illness”

“critical-illnesses”

"critically-ill"

“critical-illness”

“critical-illnesses”

ICU

ICUs

PICU

PICUs

NICU

NICUs

CICU

CICUs

“burn-unit”

“burn-units” OR

“coronary-care-unit”

“coronary-care-units”

"stepdown-unit"

“stepdown-units”

"step-down-unit"

“step-down-units”

"step-down-care"

"stepdown-care"

"step-up-unit"

“step-up-units”

"stepup-unit"

stepup-units”

"step-up-care*"

"step-up-care*"

"intermediate-care"

“intermediate-care-unit”

“intermediate-care-units”

"intermediate-unit"

“intermediate-units”

OR MeSH:

Monitoring, Intraoperative"[Mesh]

"Intraoperative Care"[Mesh]

"Intraoperative Period"[Mesh]

"Intraoperative Complications"[Mesh]

"Intraoperative Awareness"[Mesh]

"Operating Rooms"[Mesh]

"Perioperative Period"[Mesh]

"General Surgery"[Mesh]

"Surgical Procedures, Operative"[Mesh]

"surgery" [Subheading]

"Critical Care"[Mesh]

"Intensive Care Units"[Mesh]

"Critical Care Nursing"[Mesh]

"Critical Illness"[Mesh]

**Combine Concept 1 AND Concept 2 AND Concept 3**
